# The application of fluorescence techniques in meningioma surgery**—**a review

**DOI:** 10.1007/s10143-018-01062-4

**Published:** 2018-12-06

**Authors:** Bianca M. Dijkstra, Hanne-Rinck (J.R.) Jeltema, Schelto Kruijff, Rob J. M. Groen

**Affiliations:** 1 Department of Neurosurgery, University Medical Center Groningen, University of Groningen, Hanzeplein 1, P.O. Box 30.001, 9700 RB Groningen, The Netherlands; 2 Department of Surgery, University Medical Center Groningen, University of Groningen, Groningen, The Netherlands

**Keywords:** Meningioma, Fluorescence-guided surgery, Intraoperative imaging, 5-Aminolevulinic acid, Fluorescein, Indocyanine green

## Abstract

Surgical resections of meningiomas, the most common intracranial tumor in adults, can only be curative if radical resection is achieved. Potentially, the extent of resection could be improved, especially in complex and/or high-grade meningiomas by fluorescence-guided surgery using 5-aminolevulinic acid (5-ALA), indocyanine green (ICG), or fluorescein. This review aims to summarize and evaluate these fluorescence-guided meningioma surgery techniques. PubMed and Embase were searched for relevant articles. Additionally, we checked reference lists for further studies. Forty-eight articles were included in the final analysis. 5-ALA fluoresced with varying sensitivity and selectivity in meningiomas and in invaded bone and dura mater. Although ICG was mainly applied for video angiography, one report shows tumor fluorescence 18–28 h post-ICG injection. Lastly, the use of fluorescein could aid in the identification of tumor remnants; however, detection of dural tail is highly questionable. Fluorescence-guided meningioma surgery should be a reliable, highly specific, and sensitive technique. Despite numerous studies reporting the use of fluorescent dyes, currently, there is no evidence that these tools improve the radical resection rate and long-term recurrence-free outcome in meningioma surgery without neurological deficits. Evidence regarding the effectiveness and increased safety of resection after the application of these fluorophores is currently lacking. Future research should focus on the development of a meningioma-targeted, highly sensitive, and specific fluorophore.

**Electronic supplementary material:**

The online version of this article (10.1007/s10143-018-01062-4) contains supplementary material, which is available to authorized users.

## Introduction

Meningiomas are the most frequently occurring intracranial tumors in adults, accounting for one third of cases [1]. They are classified into three separate World Health Organization (WHO) grades based on histology, mitotic index, and the presence of brain invasion [2]. Although meningiomas are mostly benign and slow growing, they compress surrounding brain tissue and nerves. Depending on location and time course, symptoms such as seizures, focal neurological deficits (e.g., extremity weakness or visual changes), or (severe) mental status changes can arise. Treatment is most effective when complete surgical radical resection is achieved aiming to remove the complete tumor while preserving neurological function. Incomplete resection is a major risk factor for recurrence [2]. Currently, differentiation between meningioma tumor invasion in the dura mater or adjacent bone and reactive tissue is limited. Chances of safe gross total resection without compromising neurological outcome may decrease due to location (such as the cerebellopontine angle), invasion of dura and/or bone, and involvement of neurovascular structures. Fluorescence-guided meningioma surgery may be a helpful tool to increase the extent of resection, especially in patients with complex meningiomas.

Introduced in 1948 by Moore et al, fluorescein was applied to visualize brain tumors [3]. Later, 5-aminolevulinic acid (5-ALA) has been implemented in the field of neurosurgery, mainly in glioblastoma surgery [4]. Indocyanine green (ICG) has been utilized for video angiography mainly for neurovascular neurosurgery (aneurysms, arteriovenous malformation, and fistulas) [5]. More recently, these dyes have been applied in meningioma resections.

This review aims to give a descriptive overview of the most frequently applied intra-operative dyes (5-ALA, ICG, and fluorescein) in meningioma surgery and evaluate their role in terms of efficacy, additional extent of resection, and complication rate.

## Material and methods

PubMed and Embase were searched (search strategy in Supplemental Table 1). There were no restrictions regarding publication date; however, only full-text articles in English were assessed. The latest search update was on the 10th of July, 2018. No quality assessment was performed because the quality of the reports appeared insufficient during full-text screening of the articles.

## Results

### Literature search

**Fig. 1:**
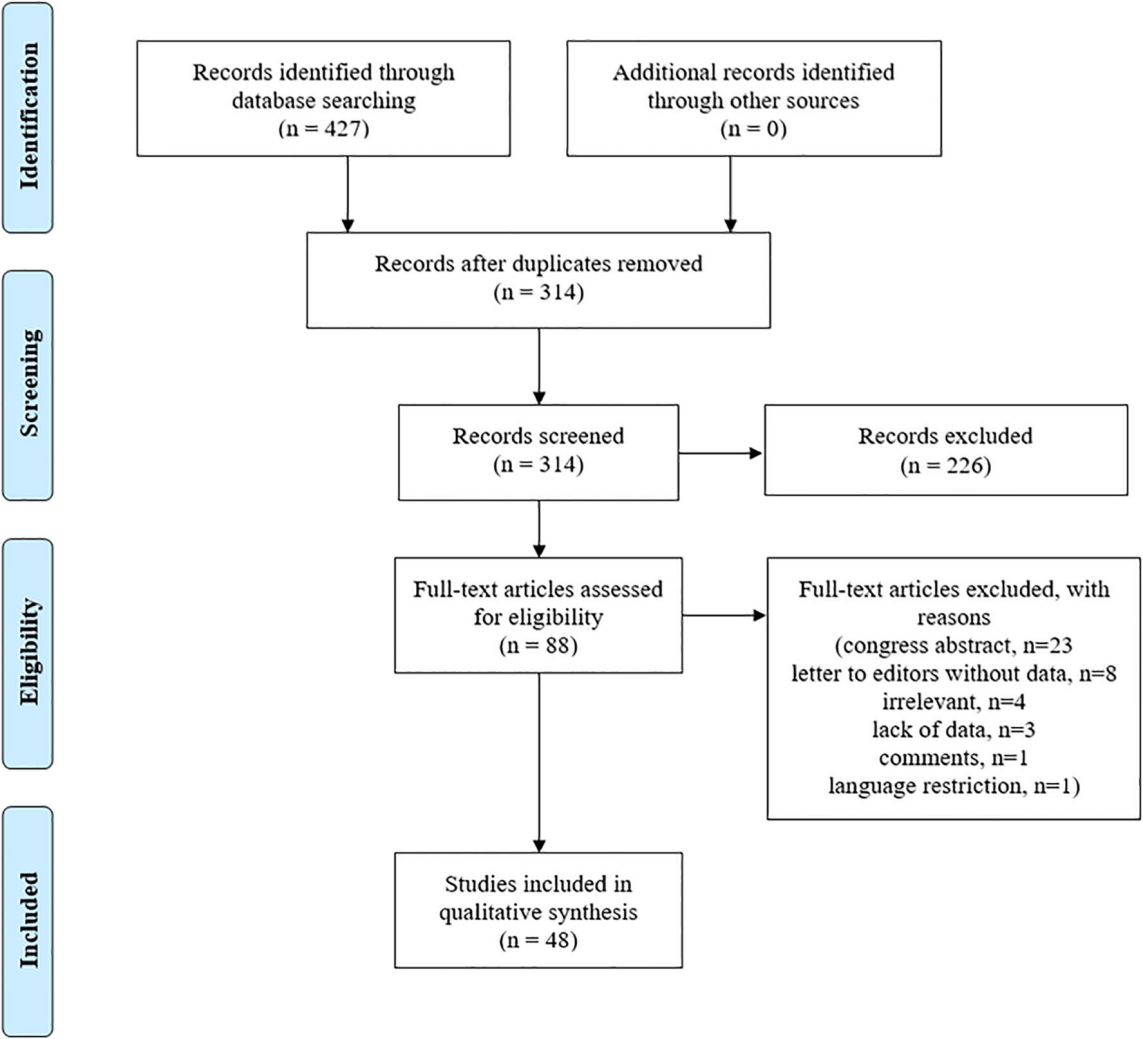
Study flow diagram

The literature search revealed 427 articles. After duplicate removal, each abstract (*n* = 314) was screened independently by two authors (BMD and RJMG) and reference lists were evaluated to identify additional relevant articles. In total, 88 full-text articles were assessed for eligibility. Of these, 48 articles were included in the final analysis. We excluded review articles, editorials, letters to editors containing no new experimental data, author replies, and comments (Fig. 1).

Below, we will consecutively describe the reported use of 5-ALA, ICG, and fluorescein in meningioma surgery. More specifically, the application of these fluorophores will be reviewed separately in imaging of the tumor, adjacent (invaded) dura mater, and bone. Additionally, other applications of these dyes will be summarized.

### 5-Aminolevulinic acid

After oral administration, 5-aminolevulinic acid (5-ALA) causes the induction of protoporphyrin IX (PpIX), a natural precursor of hemoglobin, which is normally converted to heme by ferrochelatase. In tumor cells, ferrochelatase activity is lower compared to normal tissue, and thus, PpIX accumulates mostly in tumor cells. It is excited by wavelengths of 400 nm and emits wavelengths at 620–710 nm [6]. In neurosurgery, it has been approved for glioma surgery, where it increased both the extent of resection and survival [6]. In meningioma surgery, 5-ALA has been used off-label to detect intracranial and spinal meningiomas. Its application has been described in the detection of invasion of the dural tail and bone as well (Tables 1 and 2).

**Table 1:** Types of trials of 5-ALA, ICG, and fluorescein in meningioma surgery research

Fluorophore	Tumor imaging	Dural imaging	Bone imaging	Other applications
5-ALA	X	X	X	Handheld probe, red light excitation
ICG	X			Video angiography
Fluorescein	X	X	X	Video angiography, confocal microscopy

**Table 2:** Data of studies using 5-ALA included in this review

Study	*n*	Age (years, mean)	Sex (% female)	WHO (%)	Sens (%)	Spec (%)	Histol. verific.	Focus
Kajimoto et al. 2007 [7]	24	57.1	67	I (75) II (17) III (8)	83 100 n.a.	100 67 100	Yes Yes Yes	Tumor Dural tail Bone
Morofuji et al. 2008 [8]	1	83	100	II	n.a. n.a. 100	100 100 0	Yes Yes Yes	Tumor Dural tail Bone
Eljamel 2009 [9]	2	ND	ND	ND	ND n.a.	ND 100	Yes Yes	Tumor Dural tail
Coluccia et al. 2010 [10]	33	59.7	70	I (79) I (18) III (3)	94 n.a.	100 100	Yes Yes	Tumor Bone
Bekelis et al. 2011 [11]	1	52	100	I	80 100	ND ND	Yes Yes	Tumor Probe
Whitson et al. 2011 [12]	1	53	100	II	n.a. n.a.	100 100	Yes Yes ND	Tumor Dural tail Probe
Chae et al. 2012 [13]	1	69	0	I	n.a.	100	Yes	Tumor
Della Puppa et al. 2013 [14]	3	ND	ND	ND	ND ND	ND ND	ND ND	Tumor Bone
Moriuchi et al. 2013 [15]	17	61.6	94	I (100)	ND ND	88 ND	Yes ND	Tumor Bone
Cornelius et al. 2014 [16]	31	58.7	65	I (61) II (26) III (13)	94	100	ND	Tumor
Marbacher et al. 2014 [17]	110	ND	ND	I (85) II (13) III (2)	77% fluoresced		ND	Tumor
Valdés et al. 2014 [18]	15	56.3	60	I (73) II (27)	80 94	81 ND	Yes	Tumor Probe
Wilbers et al. 2014 [19]	1	21	100	II	n.a. n.a.	100 100	Yes Yes	Tumor Dural tail
Millesi et al. 2016 [20]	204	57	69	I (76) II (16) III (8)	91% fluoresced 100% fluoresced 17% fluoresced		Yes Yes* Yes	Tumor Dural tail Bone
Potapov et al. 2016 [21]	28	56.9	79	I (86) II (14)	ND ND	ND ND	ND ND	Tumor Bone
Scheichel et al. 2017 [22]	1	78	100	II	n.a. n.a.	100 100	Yes Yes	Tumor Bone
Brokinkel et al. 2018 [23]	1	56	0	I	ND ND	ND ND	Yes Yes	Tumor Dural tail
Eicker et al. 2013 [24]	8	64.6	100	I (100)	88	100	Yes	Tumor (spinal)
Muroi et al. 2013 [25]	1	78	100	II	n.a.	100	Yes	Tumor (spinal)
Millesi et al. 2014 [26]	12	61.8	83	I (100)	100	100	Yes	Tumor (spinal)
Cornelius et al. 2013 [27]	1	65	100	I	ND	ND	ND	Bone
Della Puppa et al. 2014 [28]	12	59.8	67	I (83) II (17)	89	100	Yes	Bone
Valdés et al. 2011 [29]	6	ND	ND	I II	100	93	Yes	Probe
Cornelius et al. 2017 [30]	5	ND	ND	I (100)	ND	ND	ND	Probe
Knipps et al. 2017 [31]	13	ND	ND	I (77) II (23)	93	95	Yes	Probe
Roberts et al. 2017 [32]	6	ND	ND	I (67) II (17) III (17)	ND	ND	Yes	Red-light excitation
Total	538	58.6	81	I (79.1) II (15.9) III (4.9)				

*Only 18% histologically verified

*n*, number of relevant patients or samples; *ND*, not described

#### Tumor imaging

**Fig. 2:**
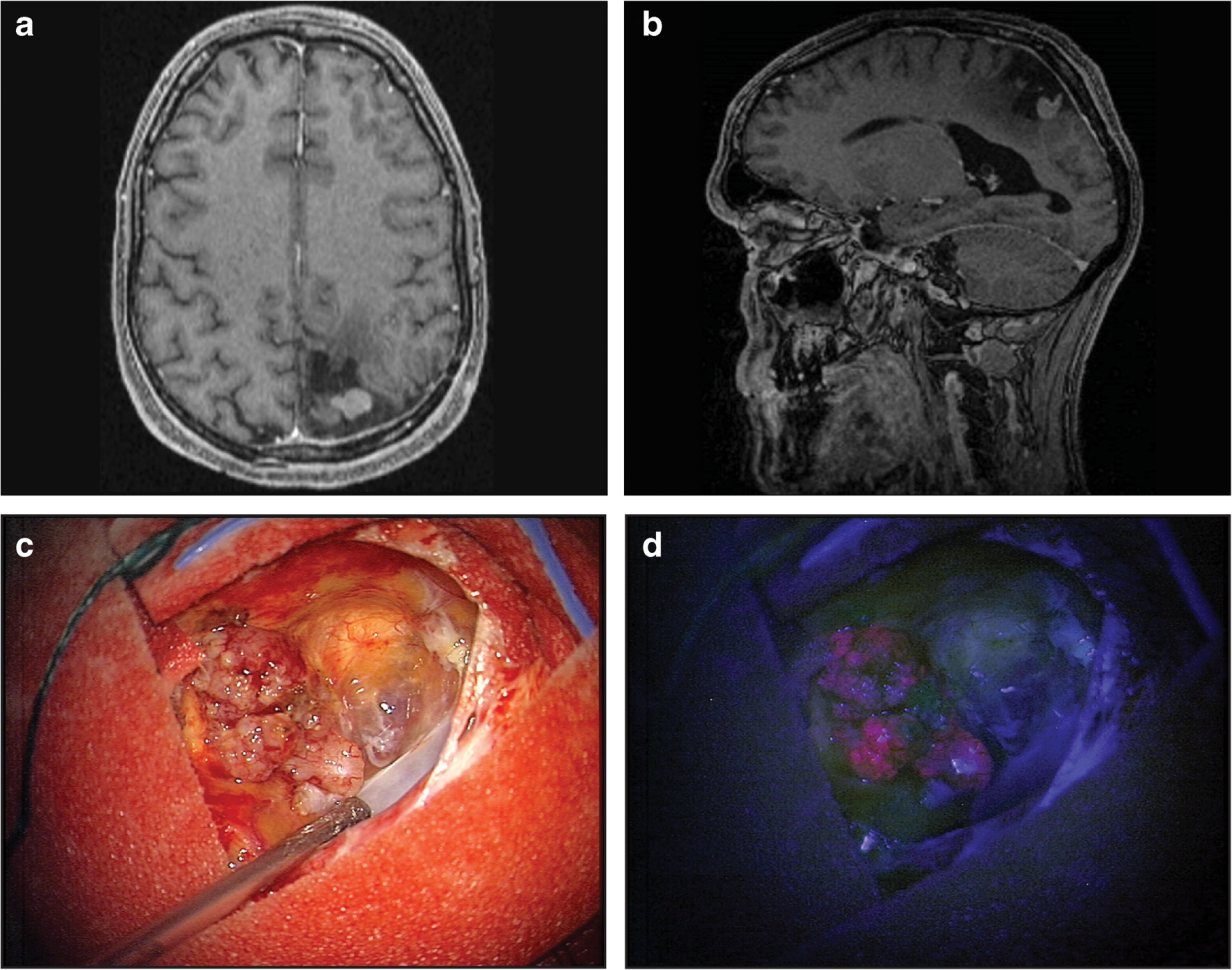
Top panel shows gadolinium contrast-enhanced MRI scans of a recurrent grade I meningioma in a 50-year-old male. Bottom panel depicts intraoperative images after preoperative oral administration of 5-ALA. MRI scans revealed a recurrent parietal meningioma (**a**, **b**). Intraoperatively, the meningioma became visible using white light (**c**) and after excitation with blue light, the meningioma showed bright violet, inhomogeneous fluorescence (**d**)

Most publications report cases or case series evaluating fluorescence status (intensity, homogeneity) in intracranial meningioma [7–23]. In a recent review [33] and meta-analysis [34], it appeared difficult to draw conclusions regarding the role of 5-ALA in meningioma surgery due to the experimental nature of 5-ALA-guided meningioma surgery. Most importantly, sensitivity and specificity rates were inconsistent between studies, although remaining high with an overall sensitivity of 92–98% and specificity of 95%. No correlation between fluorescent intensity and WHO grade or histological subtype became apparent and fluorescence was often heterogenic [7–23, 33, 34]. These findings are similar to our own experience. By way of illustration, a 50-year-old male patient underwent 5-ALA-assisted surgery of a recurrent grade I (meningotheliomatous) meningioma. Bright violet homogeneous fluorescence was observed intraoperatively with an inhomogeneous fluorescence pattern (Fig. 2).

Although 5-ALA has primarily been applied in intracranial meningiomas, its use in spinal meningiomas has been of interest as well. In two separate case reports, it allowed for the discrimination between tumor tissue and scar tissue [24] or normal tissue and residual tumor [25]. In another case series, fluorescence was homogenous in ten patients (83%) and heterogeneous in two patients (17%) [26].

#### Dural imaging

Fluorescence status in the dural tail was observed in 103 samples. Only 30 samples (29%) were histologically analyzed. Of these, six patients with histologically confirmed meningioma invasion showed fluorescence in the dural tail (20%) [7, 19]. Unaffected dura mater did not fluoresce in 17 cases (57%) [7–9, 12, 20]. Additionally, six affected dural tails (20%) did not fluoresce [20, 23]. Lastly, Brokinkel et al [23] showed PpIX formation of a non-fluorescent-invaded tail, using mass spectrometry.

#### Bone imaging

Fluorescence status in bone flaps was determined in 129 samples in total [7, 8, 10, 14, 15, 20–22, 27, 28] with subsequent histological evaluation in 114 samples (88%). Of these, 69 samples (61%) fluoresced and showed meningioma invasion upon histological examination [7, 10, 14, 20, 22, 28]. Both bright and weak fluorescence have been noted. In one report, fluorescent hyperostotic bone showed involvement in all samples (*n = 36)* and non-fluorescent non-hyperostotic bone was unaffected in all samples (*n = 5)* [28]. Invaded bone did not fluoresce in eight samples (7%) [14, 28]. Lastly, in 36 samples (32%), absence of tumor cells in non-fluorescent bone was confirmed [8, 27, 28].

#### Other applications

A handheld probe (minispectrometer) could detect fluorescence with 100% sensitivity in a spatial resolution of 1 mm^2^ in one patient harboring a skull base meningioma [11]. In another report, sensitivity of the minispectrometer was 93% (versus only 53% of surgical microscope), probably due to close proximity of the light source to the tumor [31]. Additionally, the authors reported that they could better distinguish invaded dural tail from normal dura mater using the probe [18, 29] and others claimed that they were able to differentiate glioblastomas from meningiomas based on fluorescence intensity [30].

Red light excitation of PpIX increased penetration depth up to 5 mm. Compared to blue light excitation, specificity and sensitivity were lower, and fluorescence was weaker using red light excitation. Additionally, in order to generate overlay images, this technique was not real-time. One meningioma fluoresced using blue light excitation, but did not with red light excitation [32].

### Indocyanine green

ICG was first approved for assessing hepatic and cardiac functioning, and later in ophthalmology. The use of ICG in meningioma surgery has only recently been explored, most frequently using ICG video angiography (ICG VA), visualizing the vasculature and not the tumor itself. A summary of included applications and articles has been listed in Tables 1 and 3.

**Table 3:** Data of studies using ICG included in this review

Study	*n*	Age (mean)	Sex (% female)	WHO (%)	Benefit* (%)	Focus
Lee et al. 2017 [35]	18	55.2	72	I (83) II (17)	n.a.	Tumor
Ferroli et al. 2011 [36]	14	ND	ND	ND	21 21	Arterial VA Venous VA
Kim et al. 2013 [37]	2	38.5	50	ND	100	Arterial VA
Rustemi et al. 2016 [38]	1	ND	ND	ND	100	Arterial VA
Acerbi et al. 2018 [39]	25	ND	ND	I (96) II (4)	100 100 100	Arterial VA Transdural VA Venous VA
Kim et al. 2011 [40]	15	57.3	79	I (86) II (14)	100 23	Transdural VA Venous VA
Nussbaum et al. 2012 [41]	2	62.5	50	ND	100	Transdural VA
d’Avella et al. 2013 [42]	5	58.0	80	ND	100 100	Transdural VA Venous VA
Ueba et al. 2013 [43]	2	ND	ND	ND	100	Transdural VA
Ueba et al. 2013 [44]	23**	56.9	90	I (100)	44	Transdural VA
Della Puppa et al. 2014 [45]	43	51.7	65	I (86) II (14)	85 23	Transdural VA Venous VA
Hide et al. 2015 [46]	4	ND	ND	ND	100	Transdural VA
Khurana et al. 2010 [47]	5	44.6	80	ND	20	Venous VA
Ferroli et al. 2011 [48]	2	68.5	100	ND	100	Venous VA
Total***	160	55.3	72.3	I (89.1) II (10.9)	51.8	

*Benefit of application shown in percentage

**Patient data of only ten patients has been described

***Valid percentages are shown

*ND*, not described; *VA*, video angiography

#### Tumor imaging

One publication reported the “second window technique” where ICG was administered 18–28 h preoperatively. Seventy-eight percent of cases showed tumor fluorescence. Patients injected > 22 h prior to surgery demonstrated an inverse fluorescence pattern, i.e., healthy brain fluoresced stronger than tumor. Sensitivity was high (96%), but specificity was low (39%), possibly due to camera exposure settings [35].

#### Dural imaging

No reports have established the application of ICG for the identification of meningioma invasion of the dural tail.

#### Bone imaging

No reports have established the application of ICG for the identification of meningioma bone invasion.

#### Other applications

Transdural video angiography applies ICG VA to visualize cortical veins, dural venous sinuses, and/or tumor boundaries prior to dural opening in 46 patients [39–42, 45, 46]. It allowed for a tailored dural opening without damaging underlying vasculature and the possibility of assessing vessel patency and blood flow (direction) [40–42, 45]. One group described the “Eclipse sign” [43, 44], which occurs after coagulating feeding arteries, dura mater, and middle meningeal arteries. It can be observed transdurally: the meningioma appears as a shadow after ICG injection due to reduced or absent blood flow, in contrast to dura and adjacent normal brain. This sign was observed in eight patients (40%) [43, 44]. According to the authors, absent (52%) or reduced (8%) “Eclipse sign” was suggestive of intracranial arterial supply to the tumor [43, 44].

Using arterial video angiography, patency of arteries after manipulation for tumor resection was confirmed in three patients [36]. Moreover, collateral arterial circulation was demonstrated, with subsequent sacrifice of arteries improving surgical resection without post-operative ischemia in 17 patients [36–38]. In total, 43% of 23 patients in total showed benefit from ICG VA arterial imaging, as judged by the surgeons through additional resection without ischemia.

Venous video angiography has been applied in 97 patients. According to the authors, the rate of resection was occasionally increased by confirming occlusion of veins using ICG VA in thrombosed veins [36, 40, 45, 47], identifying collateral venous drainage with [36, 39, 48] or without [39] subsequent venous sacrifice, and determining the relation of veins with the tumor (draining versus collateral veins) [39, 42, 45].

### Fluorescein

Fluorescein was first applied in 1948 for the identification of several types of brain tumor types [3]. Later it was used in ophthalmology for retinal imaging and more recently in neurosurgery, mostly in glioblastoma surgery [49]. It has a peak excitation at 490 nm and emission between 500 and 550 nm. Fluorescein has been tried in meningioma surgery with and without yellow 560 nm filter, and as a contrast agent in confocal microscopy. Reports show fluorescein imaging without appropriate controls or histological confirmation, making the evaluation of this technique extremely difficult. A summary of fluorescein intraoperative imaging is given below and an overview is listed in Tables 1 and 4.

**Table 4:** Data of studies using fluorescein included in this review

Study	*n*	Age (mean)	Sex (% female)	WHO (%)	Histol. verific.	Focus
da Silva et al. 2010 [50]*	3	ND	ND	ND	ND	Tumor
da Silva et al. 2014 [51]*	12	ND	ND	ND	ND	Tumor
da Silva et al. 2014 [52]*	9	ND	ND	ND	ND	Tumor
da Silva et al. 2014 [53]*	5	57.6	60	I (80) II (20)	Yes	Tumor, dural tail
Akçakaya et al. 2017 [54]	30	48.9	80	I (90) II (10)	Yes	Tumor, dural tail, bone, video angiography
Sanai et al. 2011 [55]	6	ND	ND	ND	Yes	Confocal microscopy
Eschbacher et al. 2012 [56]	24	ND	ND	I (83) II (17)	Yes	Confocal microscopy
Martirosyan et al. 2016 [57]	30	ND	ND	I (83) II (13) III (3)	Yes	Confocal microscopy
Total	107	50.9	77.1	I (85.4) II (13.5) III (1.1)		

*Overlap between reported patients occurs in these studies

*ND*, not described

#### Tumor imaging

The use of fluorescein-guided surgery without a microscope filter has been described in skull base [50–52] and convexity meningiomas [53]. Overlap of patients between these studies occurs. Fluorescent signal appeared in the meningioma 10 min after injection using a standard white light microscope, with heightened contrast between meningiomas and cranial nerves. Digitally quantified pre- and post-resection fluorescence showed statistical significant difference [50–52]. Using a yellow 560 nm microscope filter in 30 patients, homogeneous fluorescence occurred in 88% of meningiomas and 12% showed heterogeneous tumor fluorescence [54]. Furthermore, the authors reported that the application of fluorescein did not significantly change the surgical plan [54].

#### Dural imaging

One case series reported the identification of meningioma-positive dural tail in five patients (100%) by fluorescein fluorescence using a standard white light microscope [53]. Adjacent dura mater fluoresced in all cases (*n* = 25) applying a yellow-560 nm filtered microscope in another study, although this was not histologically confirmed [54].

#### Bone imaging

Bone involvement has not been described using fluorescein-guided standard white light surgical microscopy. However, it could be identified in five patients (20%) using filtered microscopy: bone fluorescence was observed in three bone flaps and in two skull base meningiomas [54].

#### Other applications

Confocal microscopy could reveal histopathological details, such as dense sheets, psammoma bodies, microscopic margin between tumor and brain parenchyma, and dural invasion [55, 56]. Furthermore, WHO grade and meningioma subtype could be determined correctly with high sensitivity and specificity [56, 57]. A high number of images were uninterpretable due to blood or motion artifacts and histology was vaguer compared to H&E sections [55–57].

Fluorescein video angiography at the end of resection was successfully applied to evaluate vessel patency and feeding and en passage vessels. The authors claim it was useful in vessel preservation and prevention of morbidity; however, no long-term follow-up was available [54].

## Discussion

The main risk factor for meningioma recurrence is an incomplete resection. In order to improve extent of resection, the ability to adequately identify tumor to normal tissue borders should be enhanced. This may be possible through the application of intraoperative fluorescence-guided surgery. In this review, we evaluated the literature and summarized the intra-operative use of 5-ALA, ICG, and fluorescein aiming to determine the benefit of fluorescent guidance during meningioma surgery in striving for a radical resection.

Current reports have major limitations. In this review, the majority of included studies are case reports or series with low level of evidence. Only a few report large(r) patient cohorts. To date, most studies are only anecdotal without an adequate study design: they cannot provide strong evidence and merely reflect the surgeon’s observations, e.g., extent of resection or additional safe resection. Only few reports are based on (partially) standardized protocols, for example, noting fluorescence status of tumor, bone, dura mater, and adjacent healthy tissue with at least documentation of neurological deficits and post-operative MRIs determining rate of resection. Indeed, few of the included studies followed a structured sampling approach. Thus, these outcome measures are often biased by the investigator(s) and not readily comparable between patients, even within the same study. Moreover, most studies are retrospective and no randomized controlled trials have been reported. Most reports lack reliable patient data, such as basic patient characteristics, histopathological data, and (long term) follow-up. Consequently, currently, the level of evidence for the use of fluorescence is low due to selection bias.

Reported follow-up time was mostly very limited (“post-operative days,” “post-operatively”) without long-term outcome. Short-term outcomes were mostly positive: transient or no neurological deficit and (in only a few reports) complete resection on post-operative MRI. Long-term confirmation of radical resection without neurological deficits could not be determined, e.g., in terms of progression-free survival or recurrence rates. These outcome variables are essential for the evaluation of fluorescent techniques.

Furthermore, most studies used a dichotomous scoring system to assess quality of fluorescence signal. Although this system is easily reproducible, it is not applicable for all clinical and/or experimental issues [58] and quantification of fluorescence intensity is flawed. A three- or five-level grading score would be more appropriate for the objective recording of fluorescence quality. Due to clinical applicability, the three-level system would be the first choice [58], e.g., non-fluorescent (1), minimal/moderate fluorescent (2), and highly fluorescent (3). Additionally, quantifying fluorescence using an intraoperative spectrometer enables the comparison of fluorescence intensity in a more standardized fashion by measuring absolute fluorescence levels. Although some studies applied in vivo spectrometry with quantitative analysis using an intraoperative probe, the majority only performed a qualitative analysis.

Numerous drawbacks of presently applied dyes became apparent in this review. Currently, no compelling evidence is available regarding the benefit of fluorescence-guided meningioma surgery. It might aid surgical resection in select cases through detection of meningioma remnants or invasion or by aiding in safe vascular sacrifice. Additionally, the investigated fluorophores severely lack sensitivity and specificity to distinguish meningioma (infiltration) from healthy brain tissue. Multiple studies applying 5-ALA show heterogenic meningioma fluorescent patterns [12, 33] and emphasize the role of homogenic fluorescence in complete surgical resection. However, fluorescence homogeneity was not discussed in most reports investigating any of the three investigated fluorophores. Tumor margin determination was unreliable, reducing the value of using these fluorophores. Moreover, histological analysis of individual samples was almost never or only partially reported, which diminishes the ability to correlate fluorescence (intensity) with presence of meningioma cells and severely limits the possibility of calculating sensitivity and specificity rates.

In tumor imaging, 5-ALA has high sensitivity and specificity for meningioma tissue. However, fluorescence heterogeneity and the absence of correlation between fluorescence and mitotic index remain relevant issues. Sensitivity is low(er) in dural and bone invasion compared to the fluorescence results obtained in the tumor itself. Recently, PpIX formation in a non-fluorescent-invaded dural tail was confirmed using mass spectrometry. Although emphasizing the need for a more accurate technique in fluorescence-guided meningioma surgery, this technique is highly inefficient in clinical practice. Only one study described imaging of the meningioma tumor using ICG [54]. The imaging protocol should be improved to increase sensitivity rates and tumor-to-background ratio. Other reports apply video angiography for transdural, arterial, and/or venous imaging, claiming the benefit for half of the patients, determined by surgeons personal (subjective) judgment without standardized techniques. The benefit of transdural ICG VA remains highly questionable, as established techniques such as intraoperative neuronavigation already offer accurate transdural visualization of the meningioma. In total, 75% of all investigated cases benefitted from transdural imaging following ICG VA: in the authors opinions, it increased the feeling of safety during dural opening and assessing vessel patency. Data regarding objective long-term follow-up of these “positive” cases is lacking. Authors claim arterial and venous imaging could address the uncertainty of patency, anastomotic flow, and the distinction between tumor draining veins and collateral ones. Its benefit has been described in the minority of patients, without objectifying criteria to determine superiority of this application above surgery without this tool. Fluorescein fluorescence has been performed with filter (filtered surgical microscopy) and without (standard white light microscopy). Despite the convenience and affordability of white light fluorescein surgical microscopy, contrast between tumor and surrounding tissue is markedly less evident compared to filtered surgical microscopy. Additionally, one group [54] reported that the intraoperative fluorescein-induced meningioma fluorescence did not significantly alter the surgical strategy, emphasizing the questionability of this fluorophore in meningioma surgery.

Differentiation between meningioma invasion and reactive tissue is limited at present. Pre-operatively, changes of dura mater and bone can be observed as a “dural tail sign” or bone hypertrophy. The dural tail sign is often observed on MRI scans as dural thickening adjacent to the meningioma, and is either caused by meningioma invasion or by vascular congestion, both accounting for approximately 50% of cases [59]. 5-ALA fluorescence was observed in invaded and normal dura mater, reducing sensitivity (50%). Reports describing ICG fluorescence did not determine fluorescence status of adjacent dura mater or histologically investigated dural invasion [35]. Lastly, the dura mater always fluoresces after fluorescein administration, thus making the ability to differentiate between healthy dura mater and meningioma infiltration highly questionable. This emphasizes the need for highly sensitive and specific fluorescent dyes in meningioma surgery.

Similarly, distinction between (reactive) hyperostosis and tumor invasion cannot always be readily made. Bone invasion has been reported in 20–68% meningioma patients, primarily in the presence of hyperostosis. Regardless, invasion has been described in non-hyperostotic bone as well, in 10–40% of cases [60, 61]. Indeed, all fluorescent (*n* = 21) and none of the non-fluorescent (*n* = 18) bone presented meningioma invasion in one study [28] applying 5-ALA. The only other dye showing bone fluorescence was fluorescein; however, histological results were not provided [54]. ICG bone fluorescence was not observed. In addition to location, local invasion of bone and soft tissues makes it a technical challenge to determine the tumor margin using available surgical tools. Fluorescence-guided meningioma surgery may be a helpful tool to increase the rate of resection, especially in patients with complex meningiomas.

In summary, we reviewed the current experience of 5-ALA, ICG, and fluorescein in meningioma surgery. In our opinion, fluorescence-guided meningioma surgery should be a reliable, highly specific, and sensitive technique. Despite numerous studies reporting the use of fluorescent dyes, currently there is no evidence that these tools improve the radical resection rate and long-term recurrence-free outcome in meningioma surgery and no evidence exists that safety of surgery increased due to application of these fluorescence techniques.

### Future perspectives

Failure of currently available fluorescence techniques clearly show the need for a highly meningioma-specific probe clearly distinguishing the tumor margin from healthy tissue has become apparent after analyzing current data in this review. We propose the development and intraoperative use of targeted tracers, which could be achieved by binding a meningioma-specific peptide to a fluorescent dye, preferably near-infrared due to increased penetration depth. First, this should be confirmed in in vitro cultures and subsequently in in vivo studies with mice and human subjects. As meningiomas are usually of dural origin, passage of the tracer through the blood-brain-barrier should not be an issue. The use of molecular fluorescent-guided surgery (MFGS) has been successful for various tumor types, e.g., in ovarian carcinoma [62] and peritoneal metastases of colorectal carcinomas [63] targeting αvβ3-integrin or folate receptor α and vascular endothelial growth factor α, respectively. The use of MFGS seems feasible for the optimization of meningioma surgery, which is currently a high priority in our ongoing research.

Next to this, improving and validating existing fluorescence-guided techniques could be an alternative. Current evidence is mostly based on case reports and case series and therefore an organized trial could be another next step. The organization of such a trial could improve the quality of evidence tremendously and is a necessary step in the further development of this MFGS technique. Outcome measures to be considered are among others extent of resection, fluorescence status and homogeneity, and correlation between fluorescence and WHO grade, along with long-term follow-up.

## Electronic supplementary material

ESM 1 (DOCX 13 kb)
