# Supplementary material for: The application of fluorescence techniques in meningioma surgery—a review
Source: Neurosurg Rev. 2018 Dec 6;42(4):799–809. doi: 10.1007/s10143-018-01062-4 (PMC6821664; doi:10.1007/s10143-018-01062-4)
Supplement: Supplementary file 1 — (DOCX 13 kb) [file 10143_2018_1062_MOESM1_ESM.docx]

Suppl. table 1 Search strategy in PubMed and Embase

| PubMed | Embase |
| --- | --- |
| (Meningioma[Mesh] OR Meningeal neoplasms[Mesh] OR meningioma*[tiab] OR meningeal tumor*[tiab] OR meningeal tumor*[tiab] )  AND  (Surgery, computer-assisted[Mesh] OR neurosurgery[Mesh] OR neurosurgical procedures[Mesh] OR fluoresc*[tiab] OR fluorescent guided surger*[tiab] OR fluorescent-guided surger*[tiab] OR fluorescence guided surger*[tiab] OR fluorescence-guided surger*[tiab] OR FGS[tiab] OR imaging[tiab] OR intraoperative*[tiab] OR image guided surger*[tiab] OR image-guided surger*[tiab] OR neurosurg*[tiab] OR fluorescent-guided resection[tiab] OR fluorescent guided resection[tiab] OR fluorescence-guided resection[tiab] OR fluorescence guided resection[tiab] OR surger*[tiab] OR resection*[tiab] OR fluorescence imaging[tiab] OR fluorescent imaging[tiab] OR FIGS[tiab])  AND  (Aminolevulinic acid[Mesh] OR coloring agents[Mesh] OR fluorescein[Mesh] OR fluorescent dyes[Mesh] OR indocyanine green[Mesh] OR fluorescein[tiab] OR indocyanine green[tiab] OR indocyaninegreen[tiab] OR ICG[tiab] OR 5-aminolevulinic acid[tiab] OR 5-aminolevulinic-acid[tiab] OR 5 aminolevulinic-acid[tiab] OR 5 aminolevulinic acid[tiab] OR 5-ALA[tiab] OR 5 ALA[tiab] OR ALA[tiab] OR PpIX[tiab] OR protoporphyrin IX[tiab]) | ('meningioma'/exp OR 'meningioma' OR 'meningioma*':ti,ab OR 'meningeal tumor*':ti,ab OR 'meningeal tumor*':ti,ab)  AND  ('neurosurgery'/exp OR 'neurosurgery' OR 'fluorescence imaging'/exp OR 'fluorescence imaging' OR 'image guided surgery'/exp OR 'image guided surgery' OR 'computer assisted surgery'/exp OR 'computer assisted surgery' OR 'near infrared fluorescence imaging'/exp OR 'near infrared fluorescence imaging' OR 'neurosurgery':ti,ab OR 'fluoresc*':ti,ab OR 'fluorescent guided surger*':ti,ab OR 'fluorescent-guided surger*':ti,ab OR 'fluorescence guided surger*':ti,ab OR 'fluorescence-guided surger*':ti,ab OR 'fgs':ti,ab OR 'imaging':ti,ab OR 'intraoperative*':ti,ab OR 'image guided surger*':ti,ab OR 'image-guided surger*':ti,ab OR 'neurosurg*':ti,ab OR 'fluorescent-guided resection':ti,ab OR 'fluorescent guided resection':ti,ab OR 'fluorescence-guided resection':ti,ab OR 'fluorescence guided resection':ti,ab OR 'surger*':ti,ab OR 'resection*':ti,ab OR 'fluorescence imaging':ti,ab OR 'fluorescent imaging':ti,ab OR 'figs':ti,ab)  AND  ('aminolevulinic acid'/exp OR 'aminolevulinic acid' OR 'coloring agent'/exp OR 'coloring agent':ti,ab OR 'fluorescein'/exp OR 'fluorescein' OR 'fluorescent dye'/exp OR 'fluorescent dye' OR 'indocyanine green'/exp OR 'indocyanine green' OR 'fluorescein':ti,ab OR 'indocyanine green':ti,ab OR 'indocyaninegreen':ti,ab OR 'icg':ti,ab OR '5-aminolevulinic acid':ti,ab OR '5-aminolevulinic-acid':ti,ab OR '5 aminolevulinic-acid':ti,ab OR '5 aminolevulinic acid':ti,ab OR '5-ala':ti,ab OR '5 ala':ti,ab OR 'ala':ti,ab OR 'ppix':ti,ab OR 'protoporphyrin ix':ti,ab) |
